# Reappraising Elements of the Aseptic Technique in Dermatology: A Review

**DOI:** 10.5826/dpc.1101a126

**Published:** 2021-01-29

**Authors:** Samiya Khan, Terri Shih, Shawn Shih, Amor Khachemoune

**Affiliations:** 1 Long School of Medicine, University of Texas Health Science Center at San Antonio, TX, USA; 2 David Geffen School of Medicine at University of California, Los Angeles, CA, USA; 3 University of Central Florida College of Medicine, Orlando, FL, USA; 4 Veterans Affairs Medical Center, Brooklyn, NY, USA; 5 Department of Dermatology, SUNY Downstate, Brooklyn, NY, USA

**Keywords:** gloves, sterilization, wound infection, Mohs surgery, skin neoplasms

## Abstract

Dermatologic procedures are performed under varying degrees of antisepsis, and no clear guidelines exist regarding the role of the aseptic technique in dermatology. This review aims to clarify the terminology surrounding surgical asepsis and examines the importance of various components of the aseptic technique in cutaneous surgery. Included are studies examining optimal glove type, surgical instruments, skin antisepsis, and cost-reducing protocols. Our review highlights that most dermatology procedures are not performed under completely sterile conditions due to the lack of environmental and foot traffic controls in dermatology offices. In addition, for some outpatient procedures, such as for minor excisions and Mohs surgery before reconstruction, elements of the clean technique can be used without increasing infection rates. However, data on the feasibility of a clean protocol for Mohs reconstruction is conflicting. Future prospective, randomized trials analyzing various components of the aseptic technique in dermatology are greatly needed so that guidelines can be established for practicing dermatologists.

## Introduction

Over the years, dermatologic surgical procedures have increased. Those performed in outpatient settings allow the dermatologist to provide more cost-effective treatment options for patients than procedures performed in hospital settings [1]. In fact, a survey performed by the American Society for Dermatologic Surgery showed that dermatologists performed 12.5 million procedures in 2018, a 60% increase since 2012 [2]. Despite the high volume of procedures performed by dermatologists, no standard guidelines exist regarding the extent of antisepsis required for these procedures. We aim to conduct a comprehensive literature review to elucidate our understanding of the role and necessity of the aseptic technique in dermatology practice.

## Discussion

### Reviewing the Terminology

Multiple terms that describe varying degrees of sterility during medical procedures exist. Terms such as “aseptic” and “sterile” are often used interchangeably, and multiple definitions are available. The following definitions are based on the descriptions provided by the Aseptic Non-Touch Technique Framework, a clinical practice standard developed by Rowley in the United Kingdom in the 1990s and currently implemented in more than 25 countries [3].

**Clean**: Free from visible marks and stains.

**Aseptic**: Free from pathogenic organisms in numbers needed to cause infection.

**Sterile**: Free from all microorganisms.

In addition, according to the Joint Commission, the clean and aseptic techniques are differentiated based on 4 primary factors (Table 1) [4].

#### Clean Technique

- Barrier: Appropriate hand hygiene, clean gloves.
- Patient and environmental preparation: efforts to prevent direct contamination.
- Environmental controls: routine cleaning of patient’s environment.
- Contact: sterile to sterile contact is not a consideration.

#### Aseptic Technique

- Barrier: sterile gloves, sterile drapes, and sterile masks to prevent the transfer of microorganisms from the environment to the patient.
- Patient and equipment preparation: antiseptic skin preparation of patient at time of procedure, sterile instruments, sterile equipment, sterile devices.
- Environmental controls: close doors during procedures, minimize traffic into and out of operating rooms, exclude unnecessary personnel during procedures.
- Contact: only sterile-to-sterile contact is allowed.

Historically the term sterile technique has been used interchangeably with aseptic technique. However, a true sterile technique is impossible to achieve in most healthcare settings due to the presence of airborne organisms [3].

### The Dermatology Office Environment

The practice of antisepsis in the surgical operating room involves the adoption of strict sterile techniques. Multiple, extensive guidelines have been published regarding proper sterile technique during surgery. While exact practices may vary at each institution, sterile technique in an operating room generally includes environmental cleaning, hand hygiene, preoperative skin preparation, sterile surgical attire, and maintaining a sterile surgical field. In addition, the operating room must also have high-efficiency particulate air filters and directional airflow to minimize airborne infection [5].

In contrast to the traditional operating room, dermatology offices do not have strict requirements on the use of sterile technique during dermatologic surgeries, and without ventilatory systems, achieving a truly sterile environment is impossible in dermatology offices. With a shortage of scientific data, dermatologists must personally decide for themselves the degree of sterility they would like to practice during procedures. As a result, aseptic techniques vary. A survey regarding perioperative antiseptic practices among members of the American College of Mohs Surgery showed that many traditional aseptic techniques are not used among dermatologists. For example, only 35% of respondents reported utilizing a preoperative hand scrub before Mohs Surgery [6].

When aseptic techniques are employed, they are generally used for excisional surgeries, flaps, grafts, and Mohs repairs [7]. Asepsis for these procedures is different than general surgery, however, because while sterile materials and gloves are used, there is no regulation in regard to foot traffic, airflow, or surgical scrub. Smaller procedures, such as shave or punch biopsies and curettage, use the clean technique. In addition, Mohs micrographic surgery (MMS) is typically considered a clean procedure rather than a sterile procedure because patients move between procedure and waiting rooms, covering their wound with a nonsterile bandage [8]. Despite the low incidence of strict aseptic technique use among dermatologists, the rate of surgical site infections in dermatologic surgery remain incredibly low (0.07% to 5%) [8].

### Surgical Gloves

A major area of investigation regarding aseptic practices in dermatology has been centered on the use of sterile vs clean gloves during dermatologic procedures (Table 2). In 2006, Rhinehart et al. published the first study examining this issue, conducting a retrospective chart review of 1,239 patients who underwent MMS by one of 2 surgeons. Rhinehart and his colleagues observed no statistically significant difference in surgical site infection (SSI) rates between sterile and clean gloves for MMS before reconstruction when controlling for the surgeon who performed the surgery and other components of the perioperative aseptic technique. For example, both the clean and sterile glove groups utilized identical antiseptic skin preparations, handwashing protocols, and sterile linens. In addition, if a surgical assistant was present, he or she utilized the same type of gloves as the primary surgeon [9]. Other prospective studies have similarly found clean gloves to be noninferior to sterile gloves in preventing infections [10–13].

A recently conducted systematic review of SSI rates with sterile vs clean gloves in both cutaneous and dental procedures found the same finding even when controlling for only dermatologic procedures through a subgroup of patients undergoing Mohs Surgery [14]. Another study, a prospective trial examining 3,491 dermatologic surgical procedures conducted by Rogues et al in 2007, found a lower rate of surgical site infections with sterile glove use than when sterile gloves were not used (14.7% vs 3.7%) [15]. However, this pattern was only found for reconstructive procedures such as flaps and grafts and not simple excisions. Moreover, the high rate of infection seen in this study is atypical for most dermatologic procedures and may be due to the inclusion of both hospital and outpatient procedures in this study. Based on Rogue’s analysis, it is also difficult to discern whether no gloves or nonsterile gloves were used as a control in the investigation.

Taken together, most studies show no significant difference in infection rates between sterile and clean gloves for simple outpatient dermatologic procedures. In fact, the bacterial load found on clean gloves has been found to be inadequate to cause infection [16]. On the other hand, more complex excisions and reconstructions may benefit from the use of sterile gloves; although, more prospective studies examining sterile vs clean-glove use in more complex excisions is needed.

### Surgical Instruments and Materials

The sterilization of surgical instruments is another component of the perioperative aseptic technique that helps reduce the potential of spreading infection. A few studies have been conducted regarding surgical instrument sterilization in dermatology offices. Nasseri and colleagues, for example, found that among 338 patients undergoing MMS, using a single set of sterile instruments for both the tumor extirpation and repair stages of MMS resulted in an acceptably low incidence of surgical site infections and increased cost savings [8].

Findings by Liu and colleagues further elucidate the role of the aseptic technique in dermatology by showing that new non-evidence-based guidelines proposed by the Joint Commission on the Accreditation of Health Care Organizations on the sterilization of instruments in outpatient procedures did not reduce the incidence of infection after dermatologic surgery [17]. The guidelines mandated that each item be sorted and sterilized individually but only when producer instructions were available. This increased the average time to set up a tray for each stage of the procedure, but provided no overall benefit, highlighting that evidence-based recommendations are still needed in this realm [17]. Some studies have looked at SSI rates after the use of multiple sterile instruments and materials rather than gloves in isolation. A prospective study by Rogers and colleagues in 2010 found that the use of a clean surgical technique (consisting of clean gloves, clean draping, and a reusable sterile set of instruments) in 1,000 patients for all stages of Mohs surgery (including reconstruction) led to a low rate of infection of 0.91% [18]. However, no control group was used in the study. A study published by Martin and colleagues, also in 2010, reported that heightened infection control practices reduced the rate of SSI in a statistically significant manner from 2.5% to 0.9% for 950 Mohs surgeries. The heightened aseptic regimen included jewelry restriction, alcohol hand scrub before each stage, and sterile gloves, gowns, towels, and dressings, which correspond to parts of the aseptic technique [19]. A follow-up study published in 2012 compared the SSI rates between 2 low- and high-cost infection control practices and found that the less expensive measures that omitted sterile gloves, sterile gowns, and sterile drapes did not change infection risk in Mohs surgery [20]. Based on these studies it is possible that lower cost, less sterile protocols could be implemented without compromising safety, but more specific recommendations and conclusive evidence is needed.

### Skin Antiseptics

Antiseptic scrubs are used in the aseptic technique to prepare the skin before surgery to remove transient bacteria while minimizing the remaining resident flora. Currently, no definitive guideline exists for antiseptic use in dermatologic surgery, although multiple options such as isopropyl alcohol, povidone, and chlorhexidine exist. Studies on antiseptic use in dermatology are limited, although a study by Alam and colleagues found that preoperative chlorhexidine use correlates with a lower postoperative infection risk in MMS [21]. However, the estimated absolute risk reduction was shown to between 0.45% and 0.53% and therefore exceptionally small [21]. Among studies outside the field of dermatology, a recent systematic review and meta-analysis of 19 studies concluded that chlorhexidine is associated with fewer positive skin infections compared to iodophors, such as povidone iodine and iodine povacrylex [22].

With limited data, dermatologists must use specific attributes relating to the various antiseptics to guide product usage. For example, povidone iodine is not recommended in those with an allergy to iodine, and alcohols should be avoided in wet areas of the skin due to flammability [23]. Moreover, solutions containing chlorhexidine and alcohol-containing agents should not be used on the face due to the potential for serious eye injury. For example, chlorhexidine use has been associated with irreversible keratitis leading to blindness and ototoxicity. Therefore, for head and neck procedures, one should consider povidone iodine or chloroxylenol for surgical site prep and chlorohexidine for more distant sites due to its superior length of activity [24].

### Surgical Face Masks, Caps, Gowns, and Shoes

Studies specific to the importance of surgical face masks, caps, gowns, and shoe covers—key components of the aseptic technique—are lacking. A review examining attire published by Eisen in 2011 concludes that no evidence exists to prefer one form of attire over another in dermatologic surgery and that the use of masks, head coverings, operating room shoes, and shoe covers have not been shown to avert surgical site infections [25]. For example, Cochrane Systematic Review from 2016 found no evidence that face masks reduced the rate of SSIs during clean surgery, but the limited number of studies in the review makes it unsafe to draw any definite conclusions [26]. In addition, evidence on the utility of gowns seems to be conflicting, and earlier studies analyzing bacterial load in gowns may be irrelevant due to the vastly improved gown materials of today [27]. Regarding more commonly worn outpatient attire, a systematic review by Goyal and colleagues, which included 22 studies pertaining to microbial contamination, found providers’ white coats and scrubs associated with multidrug resistant organisms, with white coats having a higher degree of contamination due to less frequent laundering [28]. Therefore, the authors suggest that white coats should be laundered at least weekly and scrubs should be changed daily. While these are general suggestions, appropriate guidelines should be established within dermatology based on the type of patient-physician encounter.

### Handwashing

Handwashing is an important component of both the clean and aseptic technique and, in the mid 1970s, was recognized as “the most important procedure in preventing nosocomial infections” by the US Center for Disease Control and Prevention (CDC) and was included in the CDC guidelines by the 1980s [29]. In the field of dermatology, the practice of proper hand hygiene is of particular concern because patient-depressed barrier function, associated with a variety of skin diseases, makes them more susceptible to infectious disease. In addition, the skin of dermatologists or their assistants can be easily contaminated. A study sampling 13 dermatologists found all physicians’ hands to be contaminated with microbial pathogens, with one hand contaminated by MRSA [30]. In the same study, compliance with handwashing was found to be only 31.4%.

Presently, the CDC recommends alcohol-based sanitation to be used predominantly, although handwashing with soap and water is preferred before eating, after using the restroom, when hands are visibly soiled, and when *Clostridium difficile* exposure is suspected [31,32]. A strict surgical hand scrub that generally includes cleaning one’s hands up to the forearm with a brush has not been tested in dermatology, and such a protocol is likely unnecessary in the dermatology practice. Nevertheless, hand antisepsis should be performed before and after patient contact.

## Conclusions

### Reappraisal of Sterile Procedures in Dermatology

The aseptic technique involves the creation of a sterile field and preservation of this sterility throughout the procedure. Due to a lack of guidelines, the degree of asepsis practiced by dermatologist varies by procedure. More complex and invasive procedures, such as Mohs reconstruction or liposuction, increase the necessity for surgical asepsis. In reality, dermatologists employ a “modified aseptic” technique when performing these procedures, as complete sterility is impossible to achieve in most dermatology offices. For example, using a single pair of sterile gloves or even a new, sterilized tray does not ensure complete asepsis because the surgical staff may not be wearing sterile attire while interacting with the patient or surgical materials. In addition, most dermatology offices see frequent foot traffic from patients and family members without regulations for when procedure room doors should be closed, and this further reduces the sterility of the environment.

Taken together, aseptic practices in dermatology vary. There is moderate evidence to show that the use of clean gloves may be justified in the setting of simple dermatologic procedures. However, this justification may not apply to more advanced dermatologic surgeries. A few studies have also shown that various cost-reducing practices, such as using a single set of sterile instruments or a clean surgical technique does not harm surgical site infection rates in dermatology. More studies are needed regarding the utility of surgical attire such as face masks, caps, and gowns in dermatology. Proper hand hygiene should, in general, always be used for both clean and aseptic procedures. While it is difficult to isolate one variable due to many possible factors leading to post-surgical infections, rigorous, well-designed randomized controlled trials are needed to help establish guidelines for the scope of asepsis required for various types of dermatologic surgery.

## Figures and Tables

**Table 1:** Definition of Terms

Term	Definition	Current Use in Dermatology
Clean technique	Maintaining an environment that is free from marks and stains Includes hand hygiene, clean gloves, efforts to prevent contamination, and routine cleaning	Punch biopsy Shave biopsy Mohs surgery
Aseptic technique	Maintaining an environment that prevents infection Includes the use of sterile attire, sterile equipment, antiseptic skin preparations, and environmental controls. Only sterile to sterile contact is allowed.	Excisions Flaps and grafts Mohs repairs

**Table 2:** Summary of Studies Comparing Sterile vs Nonsterile Glove Use in Dermatology

Author	Study Type	Findings
Rhinehart et al [9] (2006)	Retrospective	No difference in SSI rate between nonsterile or sterile gloves
Rogues et al [15] (2007)	Prospective observational	Lower rate of SSI with the use of sterile gloves compared to nonsterile gloves during reconstructive procedures No difference in SSI rate between nonsterile and sterile gloves for simple excisions
Xia et al [10] (2011)	Prospective randomized	No difference in SSI rate between nonsterile or sterile gloves
Mehta et al [11] (2014)	Prospective	No difference in SSI rate between nonsterile and sterile gloves
Brewer et al [14] (2016)	Systematic review/meta-analysis	No difference in SSI rate between nonsterile and sterile gloves (including when looking at Mohs micrographic surgery subtype)
Michener et al [12] (2019)	Prospective randomized	No difference in SSI rate between nonsterile and sterile gloves
Kemp et al [13] (2019)	Retrospective	SSI rate was 3.02% with sterile glove and 4.17% with NSG
